# Neurodevelopmental Disorders in Patients With Complex Phenotypes and Potential Complex Genetic Basis Involving Non-Coding Genes, and Double CNVs

**DOI:** 10.3389/fgene.2021.732002

**Published:** 2021-09-21

**Authors:** Martina Servetti, Livia Pisciotta, Elisa Tassano, Maria Cerminara, Lino Nobili, Silvia Boeri, Giulia Rosti, Margherita Lerone, Maria Teresa Divizia, Patrizia Ronchetto, Aldamaria Puliti

**Affiliations:** ^1^ Department of Neurosciences, Rehabilitation, Ophthalmology, Genetics, Maternal and Child Health (DiNOGMI), University of Genoa, Genoa, Italy; ^2^ Medical Genetics Unit, IRCCS Istituto Giannina Gaslini, Genoa, Italy; ^3^ Child Neuropsychiatry Unit, ASST Fatebenefratelli Sacco, Milano, Italy; ^4^ Human Genetics Laboratory, IRCCS Istituto Giannina Gaslini, Genoa, Italy; ^5^ Child Neuropsychiatry Unit, Istituto Giannina Gaslini, Genoa, Italy

**Keywords:** variants of uncertain significance, long non-coding genes, antisense gene, oligogenic disease, array-CGH, additive effect, microdeletion, microduplication

## Abstract

Neurodevelopmental disorders (NDDs) are a heterogeneous class of brain diseases, with a complex genetic basis estimated to account for up to 50% of cases. Nevertheless, genetic diagnostic yield is about 20%. Array-comparative genomic hybridization (array-CGH) is an established first-level diagnostic test able to detect pathogenic copy number variants (CNVs), however, most identified variants remain of uncertain significance (VUS). Failure of interpretation of VUSs may depend on various factors, including complexity of clinical phenotypes and inconsistency of genotype-phenotype correlations. Indeed, although most NDD-associated CNVs are *de novo*, transmission from unaffected parents to affected children of CNVs with high risk for NDDs has been observed. Moreover, variability of genetic components overlapped by CNVs, such as long non-coding genes, genomic regions with long-range effects, and additive effects of multiple CNVs can make CNV interpretation challenging. We report on 12 patients with complex phenotypes possibly explained by complex genetic mechanisms, including involvement of antisense genes and boundaries of topologically associating domains. Eight among the 12 patients carried two CNVs, either *de novo* or inherited, respectively, by each of their healthy parents, that could additively contribute to the patients’ phenotype. CNVs overlapped either known NDD-associated or novel candidate genes (*PTPRD*, *BUD13*, *GLRA3, MIR4465, ABHD4,* and *WSCD2*). Bioinformatic enrichment analyses showed that genes overlapped by the co-occurring CNVs have synergistic roles in biological processes fundamental in neurodevelopment. Double CNVs could concur in producing deleterious effects, according to a two-hit model, thus explaining the patients’ phenotypes and the incomplete penetrance, and variable expressivity, associated with the single variants. Overall, our findings could contribute to the knowledge on clinical and genetic diagnosis of complex forms of NDD.

## Introduction

Neurodevelopmental disorders (NDDs) are a heterogeneous class of conditions involving the brain, including intellectual disability and autism spectrum disorder (ASD), that affect about 1–3% of children (Miller et al., 2010). The genetics of NDDs is complex and include copy number variants (CNVs) and deleterious variants in single genes (Coe et al., 2019).

Nowadays new technologies, such as whole exome (WES) and whole genome sequencing (WGS), have become more efficient and inexpensive such that it is ongoing a debate for choosing the proper first-tier diagnostic test (Srivastava et al., 2019; Alvarez-Mora et al., 2021; Arteche-Lopez et al., 2021).

Despite of that, the detection of CNVs by the array comparative genomic hybridization (array-CGH) continues to be widely used as a first-tier test, resulting in an average diagnostic yield between 15 and 20% (Miller et al., 2010). Interpretation of CNVs is complex and, despite the use of standard classification guides (Riggs et al., 2020), most identified variants remain of uncertain significance (VUS).

Failure of interpretation of VUSs may depend on different factors, including poor annotation of protein-coding genes, lack of functional information for untranslated expressed genes or for genomic intergenic regions overlapped by the CNVs. The observation of inconsistent genotype-phenotype correlations also complicates CNV interpretation. Although most NDD causative CNVs are *de novo*, increasing evidence indicates that CNVs with high risk for NDDs show incomplete penetrance since transmission from unaffected parents to affected children has been observed. Possible explanation for the observed inconsistency of genotype-phenotype correlation may involve complex interactions of potentially pathogenic CNVs with additional/secondary CNVs, or single nucleotide variants, that could act together to determine a disease state (Velinov, 2019). According to this hypothesis, additive effects of CNVs were for instance reported in probands carrying 16p11.2 deletions and secondary CNVs that disrupt genes associated with autism and/or intellectual disability (Girirajan et al., 2010; Duyzend et al., 2016).

The goal of this study was to clarify the contribution of microdeletions and microduplications identified, by array-CGH diagnostic test, in 12 patients with complex NDD phenotype using multiple bioinformatics resources and the synergistic effort of a team of neuropsychiatrists and geneticists.

We found potential pathogenic variants in novel candidate genes for neurodevelopmental disorders. Furthermore, CNV-mediated double-hit mechanisms seem to play a relevant role in the complex forms of neurodevelopmental disorders affecting our patients. Indeed, these co-occurring hits involved already known NDD-associated genes or hypothetical novel NDD candidate genes enriched for pathways or biological processes known to be implicated in neurodevelopment.

## Methods

### Clinical Assessment

The clinical assessment of the patients comprised a thorough medical history also attentive to investigating systemic symptoms and sleep, neurological-behaviour examination, developmental/cognitive assessment, and basic metabolic screening. Prior to array-CGH analysis, all patients were first evaluated by using different genetic tests for the common genetic causes of NDDs, including karyotype analysis, and tests for Fragile X syndrome. Written informed consent was obtained from the patient’s parents or legal representative. This study was reviewed and approved by the Ethics Committee of the Italian Regione Liguria (R. P. 001/2019).

### CNV Detection and Annotation

Array-CGH analysis was performed on DNA samples, extracted from peripheral blood, using a whole-genome 180 K Agilent array with ∼13 Kb overall median probe spacing (Human Genome CGH Microarray, Agilent Technologies, Santa Clara, CA, USA). Data were analyzed using Agilent CytoGenomics and genomic positions reported according to the human genome assembly (GRCh37/hg19). All detected CNVs were tested for inheritance by hybridization of the parental DNA with the same array platform. *De novo* CNVs were confirmed either by FISH analysis or by quantitative real-time PCR as already reported (Vaccari et al., 2014; Tassano et al., 2015). To assess the clinical significance of the detected CNVs, we used several tools. Aberration segments were reviewed using GRCh37 hg19 of UCSC Genome Browser (http://genome.ucsc.edu/index.html). We annotated all detected CNVs and CNV-encompassed genes across public databases: Genomic Variants Database (DGV) (http://dgv.tcag.ca/dgv/app/home), DECIPHER (https://decipher.sanger.ac.uk/), Clinical Genome Resource (ClinGen) (https://clinicalgenome.org/), the Online Mendelian Inheritance in Man (OMIM) (http://www.omim.org), Simons Foundation Autism Research Initiative (SFARI) database (https://gene.sfari.org/), databases of mouse models (Mouse Genome Informatics, MGI) (http://www.informatics.jax.org). Literature mining was also performed. We considered only variants present in <0.1% of control individuals. We checked CNVs for the presence of dosage-sensitive genes (haploinsufficiency/triple sensitivity score) by using Clinical Genome Resource (ClinGen) consortium resource (https://dosage.clinicalgenome.org/). We also considered probability of intolerance to loss-of-function (LoF) variants (pLI) as reported in the Genome Aggregation Database (gnomAD, (https://gnomad.broadinstitute.org/). Genes with pLI scores of 0.9 or higher are extremely intolerant to heterozygous LoF variation, and thus haploinsufficient (Lek et al., 2016).

We then investigated deleted/duplicated regions for encompassing topologically associating domains (TADs), or their boundaries (TDBs), using Hi-C (http://3dgenome.fsm.northwestern.edu/). Indeed, TADs data from genome-wide higher order chromatin interaction data in human embryonic stem cells (h-ESC), h-ESC derived neural progenitor cells (H1NPC) (Dixon et al., 2015), and brain cortex (Schmitt et al., 2016), were downloaded (http://chromosome.sdsc.edu/mouse/download.html) (see Supplementary file) and mapped to hg19 coordinates using the UCSC browser. We then investigated identified CNV-genes, not previously associated with genetic diseases, for relevance to NDDs by considering different criteria: expression in the brain; reported mutations in the mouse associated with a neurodevelopmental phenotype; interaction with genes known to be associated with a neurodevelopmental disorder. To identify CNV disrupted genes with high brain expression, we used the BrainSpan database (http://www.brainspan.org/static/download.html) (Huckins et al., 2019) and extracted the list of genes with average log2 RPKM >4.5 (the top 18%), as also reported (Addis et al., 2018; Cerminara et al., 2021). We further investigated brain expression of genes at single gene isoforms using GTEx data (https://www.gtexportal.org/home/). We searched for gene-specific mouse models presenting a neurodevelopmental phenotype using MGI database. CNV-genes coding for microRNAs (miRNAs) were further investigated to detect the presence, among their possible targets, of any genes known to be associated with NDDs (Supplementary file) or to be involved in brain development and/or function by using miRDB (http://mirdb.org/), Target Scan (http://www.targetscan.org/vert_72/) and DIANA TOOLS (http://diana.imis.athena-innovation.gr/DianaTools/index.php).

### Network Analysis of Candidate Genes

We used GeneCodis4 (Tabas-Madrid et al., 2012) to unveil enrichment of annotations, as already reported (Cerminara et al., 2021). Briefly, all the genes obtained from either duplicated or deleted regions were used as input in GeneCodis4 together with known NDD-associated genes, as those related to ASD and reported in SFARI database and those reported in OMIM as associated with intellectual disability (see Supplementary file). This tool allows the classification of genes according to their putative biological function by screening the Gene Ontology (GO), OMIM, Panther, and KEGG Pathways. In the analysis, the hypergeometric test was applied followed by the false discovery rate correction (FDR) with a cut-off of 5% to determine which annotations were significantly enriched. For GO analysis, various hierarchical levels of the annotation data structure were used. All genes of the top gene ontology terms, including candidate and known NDD genes, were then projected onto the STRING network (v11) (Szklarczyk et al., 2019). Edges within the STRING network were thresholded at 0.4, according to the authors’ recommendation. A graphical representation of the GeneCodis4 and STRING results was obtained by using Cytoscape tool (Shannon et al., 2003).

### Pathogenicity Evaluation and Graphical Representation of CNVs

To evaluate the pathogenicity of variants we focused on the following criteria: 1) patients with multiple CNVs, enclosing NDD genes, which could have additive effects on the patients’ phenotype on the basis of results from gene enrichment and protein-protein interactions analyses; 2) patients with CNVs encompassing regions that control the expression of known NDD genes, including those with potential long-range effects; 3) patients whose CNVs encompass genes not yet reported as NDD-associated, but whose expression profile and function suggest a possible involvement in the disease, when at least another patient is reported with a similar CNV and phenotype in publically available databases. Results of this analysis were graphically reported using UCSC and specific Custom Tracks. In particular we used RefSeq Curated for gene representation and OMIM genes to underline genes already associated with disease. Decipher track was manually modified; we selected patients with CNVs similar to those of our patients, that is either deletions or duplications according to those found in our patients and overlapping approximately the same region and genes. We selected Decipher CNVs only if their related patients had a NDD phenotype (we kept out patients for whom we had no information about their phenotype). The CNVs thus selected were further investigated for being present in the same patient with other secondary CNVs overlapping additional NDD genes, and in this case reported in figures (marked with letters). Developmental Delay case track includes manually selected CNVs similar in size and gene content to those of our cases; we proceeded analogously also for Developmental Delay Control CNVs. A track reporting SFARI genes was added to underline the presence of a known ASD associated gene in the genomic region shown in figures. Brain-expressed gene track represents genes with a prevalent expression in the brain according to the BrainSpan data processed as detailed above, useful to highlight possible new NDD candidate genes.

## Results

### Clinical Presentation of Patients

The 12 patients we report were all born to non-consanguineous parents. After clinical evaluation and follow up, the employment of specific tests and instruments, all subjects showed a complex phenotype, each of them characterized by different neurological features and other associated impairments and comorbidities. The main clinical features were represented by a variable degree of intellectual disability, in three cases associated with a diagnosed ASD, and epilepsy. Language disorder, self-aggressive behaviours and sleep disturbances could be also observed (see Table 1).

**TABLE 1:** Clinical features.

Patient ID	ASD	ID	Other NDDs	Neurological signs/disorders	Others	Results of other laboratory tests
IGGAC01	NO	ID	Language disorder, Coordination disorder, Atypical behaviours, Attention disorder and Hyperactivity, Self-aggressive behaviours	Hypotonia Epilepsy, Sleep disturbances	Gastrointestinal disorders, dysphagia and selective feeding	Normal blood amino acid and organic acids levels
						Fragile X analysis: negative
IGGAC04	NO	ID	NO	Hypotonia and Epilepsy	Prenatal gastrointestinal perforation	Normal blood amino acid, organic acids levels, acylcarnitine profile, guanidinoacetic acid
						Karyotype, Fragile X analysis: negative
IGGAC06	YES	ID	NO	Sleep disturbances	Congenital lymphedema	Normal blood amino acid and organic acids levels
						Karyotype, Fragile X analysis: negative
IGGAC07	NO	ID	Atypical behaviours, Attention disorder and Hyperactivity	NO	NO	Findings of the amniocentesis
IGGAC08	NO	Borderline	Language disorder, Attention disorder and Hyperactivity	NO	Growth defect	Normal blood amino acid and organic acids levels
						Fragile X analysis: negative
IGGAC10	NO	ID	NO	Epilepsy	NO	Normal blood amino acid and organic acids levels
						Fragile X analysis: negative
IGGAC13	YES	ID	NO	Epilepsy	NO	Normal blood amino acid and organic acids levels
						Karyotype, Fragile X analysis: negative
IGGAC14	NO	ID	Learning disorder and Aggressivity	Chiari I malformation and Mild skull base abnormality	NO	Normal blood amino acid and organic acids levels
						Karyotype, Fragile X analysis: negative
IGGAC15*	NO	Borderline	Psychomotor delay and mild Atypical behaviours	NO	NO	Fragile X analysis: negative
IGGAC16*	NO	ID	Hyperactivity	Sleep disturbances	NO	Fragile X analysis: negative
IGGAC023	YES	ID	NO	Enuresis	NO	Normal blood amino acid and organic acids levels
						Karyotype, Fragile X analysis: negative
IGGAC024	NO	ID	Language disorder and Hyperactivity	NO	Growth defect, Brain midline defects	Normal blood amino acid and organic acids levels
						Karyotype, Fragile X analysis: negative

ASD, autism spectrum disorder; ID, intellectual disability. * Brother and sister.

### Additive Effects of CNVs Involving Known NDD Genes

Four patients presented with two CNVs, overlapping known NDD genes, and inherited, respectively, by each of their healthy parents. We thought that in these cases, CNVs could additively contribute to the patient’s phenotype (Table 2).

**TABLE 2:** Overview of the CNVs found in the patients.

Patient ID	Gender	CNV coordinates (hg19)	Minimal region (Kb)	del/dup	Inh	Total genes (RefSeqAll)	Candidate genes
IGGAC13	M	7:146464283-146532437	68	del	mat	1	*CNTNAP2*
		11:40303114-40694847	392	del	pat	1	*LRRC4C*
IGGAC14	M	17:76419867-76548866	129	del	pat	3	*DNAH17*
		X:561527-908212	347	dup	mat	1	*SHOX*
IGGAC15^a^	M	15:22765628-23208901	443	del	mat	6	*NIPA1,CYFIP1,TUBGCP5,NIPA2*
		6:33337684-33669083	331	dup	*de novo*	13	*SYNGAP1*
IGGAC16^a^	F	15:22765628-23208901	443	del	mat	6	*NIPA1,CYFIP1,TUBGCP5,NIPA2*
		6:33337684-33669083	331	dup	*de novo*	13	*SYNGAP1*
		16:6371455-6568176	197	del	pat	1	*RBFOX1*
IGGAC07	M	12:108487109-108896252	409	dup	mat	3	*WSCD2*
		14:22360671-23120435	760	dup	*de novo*	5	*ABHD4*
		22:22998284-23643223	645	dup	mat	8	*GNAZ*
IGGAC10	M	3:123113523-123997093	884	dup	pat	9	*ADCY5*
		6:86338564-86413285	75	del	mat	4	*SYNCRIP*
IGGAC06	M	9:10286927-11086155	799	del	mat	2	*PTPRD*
		11:116182875-116677043	494	del	not mat	4	*BUD13*
IGGAC08	M	4:175734964-175934265	199	del	mat	3	*GLRA3*
		6:140767462-141008265	241	del	*de novo*	1	*MIR4465*
		10:134289353-134917702	628	dup	*de novo*	9	*INPP5A*
IGGAC04	M	5:88232587-90181774	1,949	del	*de novo*	9	*MEF2C-AS1* and *ADGVR1*
IGGAC01	F	16:8969984-10078802	1,109	dup	*de novo*	6	*USP7* and *GRIN2A*
IGGAC24	M	19:55948706-56471996	523	dup	*de novo*	24	*SHISA7*
IGGAC23	M	10:125121038-127119447	1,998	dup	*de novo*	13	*CTBP2*

a Brother and sister.

**FIGURE 1:**
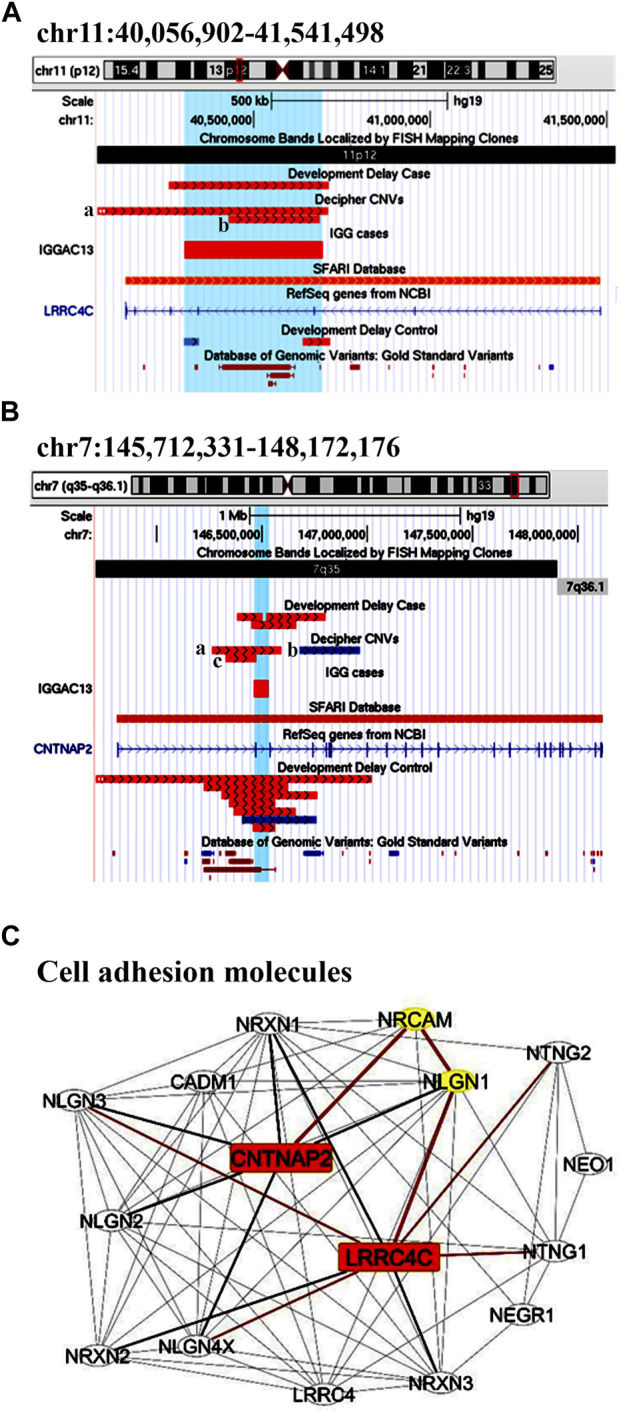
Additive effects between *CNTNAP2* and *LRRC4C* in patient IGGAC13. **(A**) Screenshots of chromosome 11 region overlapping the deletion of patient IGGAC13 (red bar IGGAC13, chr11:40,303,114-40,694,847). Decipher CNVs (a: deletion in patient 282991; b: deletion in patient 276911). **(B)** Screenshots of chromosome 7 region overlapping the deletion of patient IGGAC13 (red bar IGGAC13, chr7:146,464,283-146,532,437). Decipher CNVs (a: deletion in patient 319381; b: intragenic duplication in patient 255117; c: deletion in patient 250646). Only Decipher patients with additional CNVs that overlap neurodevelopmental genes were selected and reported in the figure. **(C)** The network depicts the protein-protein interactions (grey lines), experimentally determined interaction (red lines) and co-expression (bold black lines) of the KEGG pathway “Cell adhesion molecule,” revealed by STRING analysis on the basis of enrichment obtained with GeneCodis4, and visualized by Cytoscape tool. The Figure shows the clustering of the candidate genes with other known NDD genes. In yellow the interacting molecules directly linking the two candidate genes to each other.

The first case**,** IGGAC13, is a male patient with a maternal deletion of chromosome 7 involving *CNTNAP2* and a paternal deletion of chromosome 11 involving *LRRC4C* (Figures 1A,B). Both genes are present in the SFARI database. Few CNVs are reported to overlap these two genes in control individuals and in NDD patients according to Developmental Cases and Decipher databases. Of note, NDD patients reported in Decipher and represented in Figure 1 carried one additional CNV involving other NDD-associated genes. Indeed, a case has been previously reported with two deleterious variants, one of which disrupting *LRRC4C* and one affecting another SFARI-reported gene *DPP6* (Maussion et al., 2017). The two variants, as in our case, were inherited one from the mother and one from the father, both unaffected.

*CNTNAP2* encodes a member of the neurexin family which functions in the nervous system as cell adhesion molecule. *LRRC4C* (NGL1) is a specific binding partner for netrin G1, which is a member of the netrin family of axon guidance molecules. In fact, both molecules, *CNTNAP2* and *LRRC4C,* are implicated in synapse formation (Um and Ko, 2017). We hypothesized that, while half dosage of each of these two molecules is compatible with a normal development, half dosage of both genes can impair synapse correct development and function. In fact, gene enrichment and String analysis showed that both genes are members of the KEGG cell adhesion molecule pathway and interact with other known NDD genes (Figure 1C; Supplementary Table S1).

**FIGURE 2:**
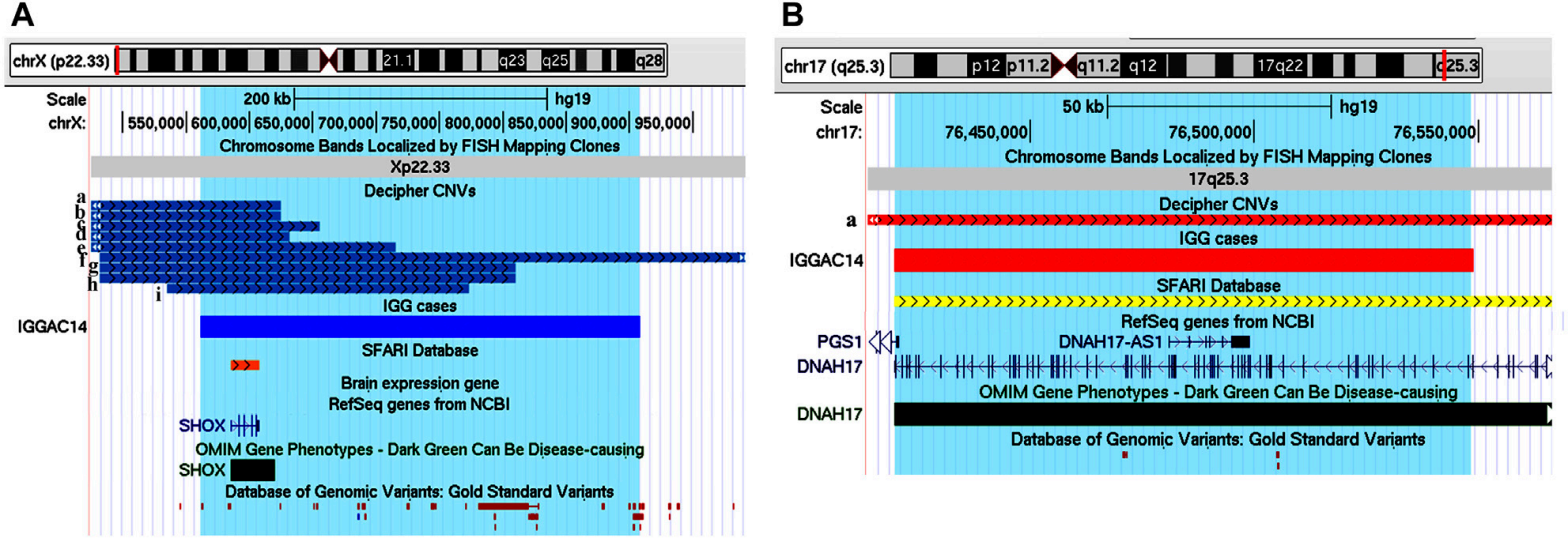
Additive effects between *SHOX* and *DNAH17* in patient IGGAC14. **(A)** Screenshot of chromosome X region overlapping the duplication of patient IGGAC14 (blue bar, chrX:561,527-908,212). Duplications in Decipher database (a: patient 259967; b: patient 367079; c: patient 380394; d: patient 284815; e: patient 289670; f: patient 322776; g: patient 367150; h: patient 279033; i: patient 353723). **(B)** Screenshot of chromosome 17 region encompassing the deletion of patient IGGAC14 (red bar, chr17:76,419,867-76,548,866). Deletions in Decipher database (a: patient 289758).

The second case is a male patient (IGGAC14) that inherited from his healthy father a deletion of chromosome 17 encompassing a SFARI gene, *DNAH17,* and its antisense gene *DNAH17-AS1*. *DNAH17* encodes a heavy chain associated with axonemal dynein, a member of microtubule-associated motor protein complexes. From his healthy mother, the patient inherited a duplication involving *SHOX* on chromosome X (Figure 2), also reported in the SFARI database**.** *SHOX* belongs to the paired homeobox family and is located in the pseudoautosomal region 1 (PAR1) of X and Y chromosomes. Enrichment of *SHOX* microduplications in NDD cases, particularly in those with ASD, has been described (Tropeano et al., 2016). Gene enrichment evidenced a function of *SHOX* in the regulation of transcription by RNA polymerase II, and of *DNAH17* in microtubule-based movement, two important biological processes in neurodevelopment disorder (Figure 2; Supplementary Table S1). As shown in Figure 2A, other patients with NDD phenotype are reported in Decipher database to have duplications of *SHOX* and a secondary CNV involving neurodevelopmental genes. Indeed, a chromosome 17 deletion similar to that of our patient was reported in Decipher database in a case with NDD phenotype having a secondary CNV also overlapping developmental genes (Figure 2B).

**FIGURE 3:**
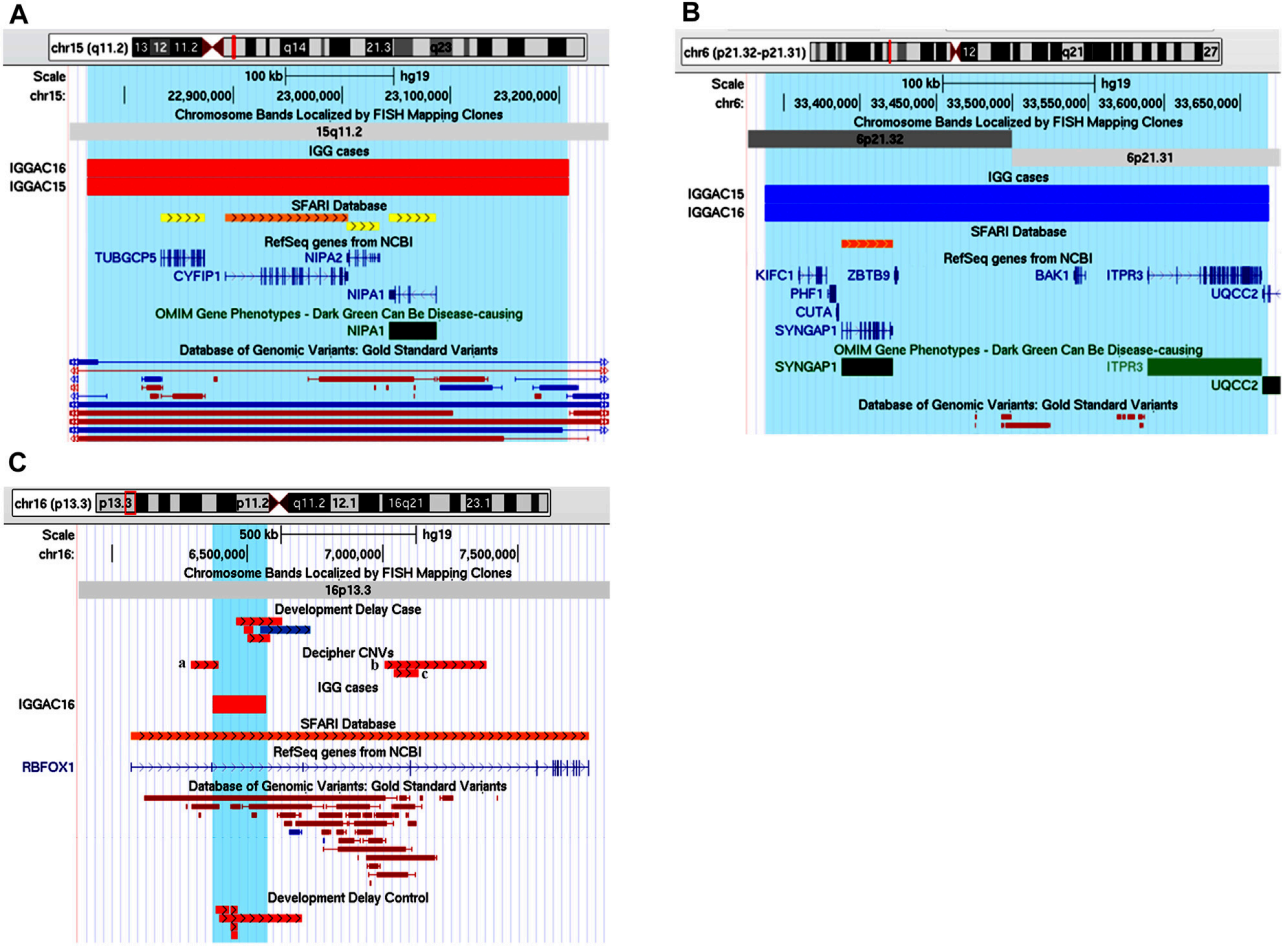
Additive effects in a familial case, siblings IGGAC15 and IGGAC16. **(A)** Screenshot of chromosome 15 region encompassing the deletion shared by the two siblings (red bars, chr15:22,765,628-23,208,901). **(B)** Screenshot of chromosome 6 region encompassing the duplication shared by the two siblings (blue bars, chr6:33,337,684-33,669,083). **(C)** Screenshot of chromosome 16 region encompassing the deletion of patient IGGAC16 (red bar, chr16:6,371,455-6,568,176). Deletions reported in Decipher patients (a: patient 283262; b: patient 327250; c: patient 403649).

We observed a family with two affected siblings, a male (IGGAC15**)** and a female (IGGAC16**)**, showing discordant phenotypes. They inherited from their unaffected mother a deletion encompassing a syndromic region at 15q11.2 (Figure 3A). The siblings also shared a duplication involving the SFARI gene *SYNGAP1* (Figure 3B). Of note, the *SYNGAP1* duplication was not observed in parents, at least in their blood-extracted DNA, thus leading to suppose mosaicism in either father or mother. Patient IGGAC16 also inherited a paternal, recurrent deletion involving the SFARI gene *RBFOX1* (Figure 3C). Interestingly, loss of function variants of *SYNGAP1* have been identified in patients with ASD and intellectual disability with or without epilepsy. However, to the best of our knowledge, no cases with duplication or gain of function variants have been reported so far. Overexpression of SynGAP was reported to block neurite outgrowth by a mechanism that involves Ras-like GTPase cascade (Tomoda et al., 2004), to produce a depression of AMPAR-mediated excitatory postsynaptic currents in neurons (Rumbaugh et al., 2006), and to regulate decreasing and increasing miniature excitatory synaptic currents in hippocampal neurons (McMahon et al., 2012). In fact, some phenotypic differences between the two siblings were observed. The male patient showed a mild phenotype characterized by a borderline cognitive functioning associated with atypical behaviours and psychomotor delay in early infancy, his sister had a more complex phenotype with mild cognitive impairment, irregular sleep rhythms, hyperactivity, coordination, and language disorders. We could speculate that the two CNVs shared by the two siblings can explain the phenotypic features shared by both of them, while the third CNV could have worsened the phenotype of the female patient.

### Addictive Effects of CNVs Involving Potential Novel Candidate Genes and New Pathogenic Mechanisms

Four patients reported double CNVs involving genes or genomic regions not yet described as NDD-associated but that could act in concert, similarly to the four cases described above, with a potential impact on the patients’ phenotype (Table 2).

**FIGURE 4:**
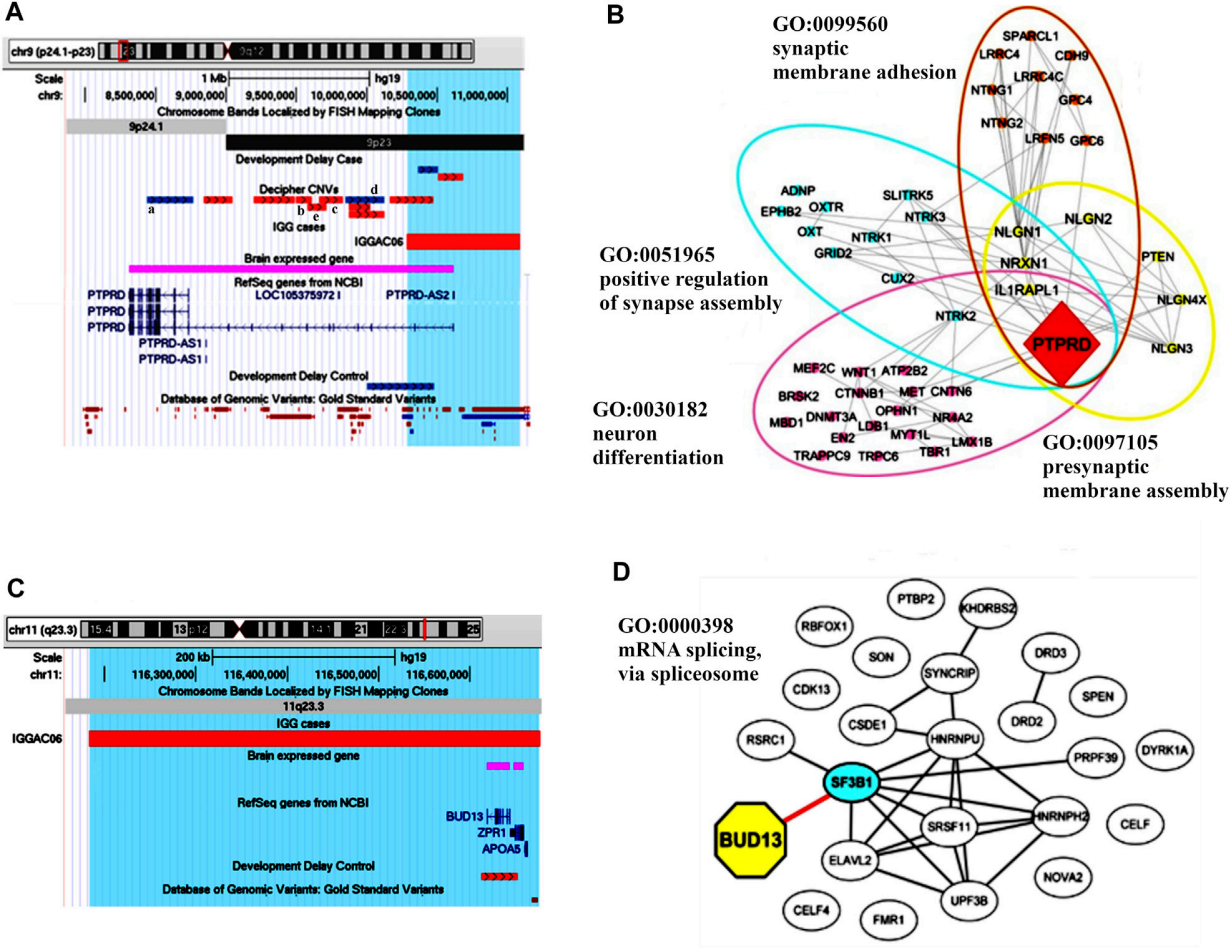
Additive effects between *PTPRD* and *BUD13* in patient IGGAC06. **(A)** Screenshot of chromosome 9 region overlapping the deletion of patient IGGAC06 (red bar, chr9:10,286,927-11,086,155). Decipher CNVs (a: intragenic duplication in patient 333436; b: deletion in patient 412118; c: deletion in patient 337260; d: intragenic duplication in patient 260089 and e: deletion in patient 279686). **(B)** The network depicts the protein-protein interactions (grey lines) revealed by STRING analysis (visualized by Cytoscape tool) on the basis of the enrichment obtained with GeneCodis4 tool. The Figure shows the enriched GO terms clustering the candidate gene *PTPRD* and known NDD genes. **(C)** Screenshot of chromosome 11 region overlapping the deletion of patient IGGAC06 (red bar, chr11:116,182,875-116,677,043). **(D)** The network depicts the protein-protein interactions (grey lines) and experimentally determined interaction with a SFARI gene (red line) revealed by STRING analysis (visualized by Cytoscape tool) on the basis of the GO term “mRNA splicing *via* spliceosome”. The network indicates a direct interaction of *BUD13* with the known NDD gene *SF3B1*.

The first case (IGGAC06) carried a maternal deletion of chromosome 9 and a non-maternal deletion of chromosome 11; as genetic analysis of the patient’s father was not available, we cannot determine if the chromosome 11 deletion was *de novo* or paternally inherited (Figures 4A,C). The chromosome 9 deletion encompassed *PTPRD*, which encodes a protein-tyrosine phosphatase receptor, and *PTPRD-AS2*, a long non-coding RNA (lncRNA) gene known as *PTPRD* antisense RNA 2 (head-to-head) predicted to make expression of mRNA more stable and thus enhancing *PTPRD* protein production. Thus, a reduced expression of *PTPRD* can be hypothesized because of the deletion of the first two untranslated exons of the gene and of *PTPRD-AS2*. A total of 7 deletions encompassing exonic regions of *PTPRD* are reported in controls, while several CNVs and, in particular, 16 deletions and one intragenic duplication involving coding regions of the gene are present among cases in Developmental Delay and Decipher databases with NDD phenotypes. Five among Decipher cases have an additional CNV involving brain expressed genes that, analogously to those reported in patient IGGAC06, could have an additive effect in causing patient’s phenotype. Interestingly, *PTPRD* is predicted to be intolerant to loss of function variants (pLI = 1) and to haploinsufficiency mechanisms (score = 0.75; value above 0.5 are predicted to be haploinsufficient) (Shihab et al., 2017). *PTPRD* has a role in synaptic adhesion and synapse organization and can bidirectionally induce pre- and postsynaptic differentiation of neurons by trans-synaptically binding to interleukin-1 receptor accessory protein (*IL1RAP*) (Yoshida et al., 2012; Yamagata et al., 2015). In the mouse, *PTPRD* was shown to regulate neurogenesis (Tomita et al., 2020) and mice lacking *PTPRD* showed impaired learning with enhanced hippocampal long-term potentiation (Uetani et al., 2000), thus suggesting a role for *PTPRD* in neurodevelopmental disorders. In OMIM database, *PTPRD* is not associated with a disorder, and its implication in human disease is still to be completely clarified. Homozygous deletions of *PTPRD* were observed in a patient with intellectual disability and trigonocephaly (Choucair et al., 2015) and his family members carrying the same deletions were reported to be unaffected. Instead, a heterozygous *de novo* splicing variant of the gene was reported in a girl with moderate nonsyndromic developmental delay (Yan et al., 2019). Furthermore, genetic studies showed an oligogenic association of *PTPRD* variants with obsessive-compulsive disorder (Mattheisen et al., 2015) and with restless leg syndrome (Schormair et al., 2008). Overall, results from various genetic studies are consistent with both major/oligogenic and modest/polygenic contributions of common and rare *PTPRD* variations in neurological, behavioural, and neurodevelopmental disorders (Uhl and Martinez, 2019). Gene enrichment and String analysis showed *PTPRD* interacting with many NDD genes playing a role in synaptic membrane adhesion and neuronal development (Figure 4B; Supplementary Table S1).

The chromosome 11 deletion encompassed *BUD13,* which encodes a component of the retention and splicing (RES) complex of the spliceosomal complex. In zebrafish, the lack of *BUD13* caused defects in intron splicing specifically in genes with neurodevelopmental regulatory functions, thus resulting in a decrease of differentiated neurons and brain developmental defects (Fernandez et al., 2018). GeneCodis analysis showed an enrichment of *BUD13* with various NDD genes in the GeneOntology category of mRNA splicing, via spliceosome, a biological process with an important role in neurodevelopment (Figure 4D, Supplementary Table S1).

**FIGURE 5:**
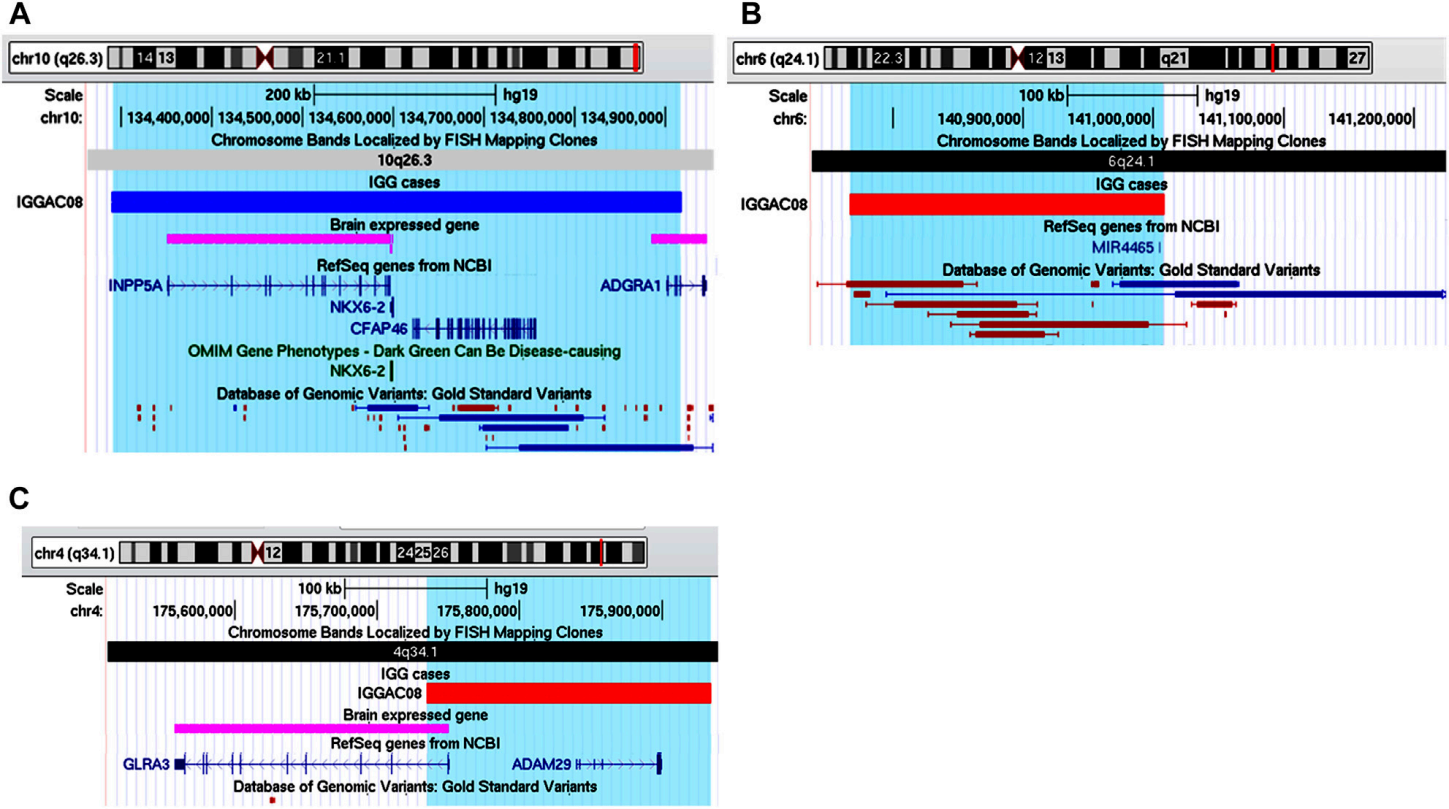
Additive effects of *INPP5A*, *MIR4465* and *GLRA3* in patient IGGAC08. **(A)** Screenshot of chromosome 10 region encompassing the duplication of patient IGGAC08 (blue bar, chr10:134,289,353-134,917,702). **(B)** Screenshot of chromosome 6 region encompassing the deletion of patient IGGAC08 (red bar, chr6:140,767,462-141,008,265). **(C)** Screenshot of chromosome 4 region encompassing the deletion of patient IGGAC08 (red bar, chr4:175,734,964-175,934,265).

Patient IGGAC08 has a complex phenotype mainly characterized by language disorder, hyperactivity, learning difficulties, borderline cognitive level, motor skill impairment, and short stature (Table 1). His mother also had school difficulties and showed dyslexia in childhood. Mother and son share a deletion of chromosome 4q34.1 encompassing *GLRA3* which encodes the alpha-3 subunit of the neuronal glycine receptor, a ligand-gated ion channel. In addition, patient IGGAC08 had a *de novo* deletion involving *hsa-mir-4465* and a *de novo* duplication including *INPP5A* (Table 2 and Figures 5A–C). *GLRA3* receptors are expressed in spinal cord, brainstem, hippocampus, amygdala, striatum, and cortex. The glycine receptor is a glycoprotein composed of 5 subunits, three α and two β subunits. The alpha subunits bind the glycine ligand while the beta subunits bind to gephyrin, a cytosolic protein required for a regulated synaptic aggregation and clustering of these receptors.

Gephyrin has functional links with several synaptic proteins, mutations of which have been reported in various neurodevelopmental disorders (Choii and Ko, 2015; Kim et al., 2021). Half dosage of *GLRA3* may contribute to impair gephyrin-mediated aggregation and post-synaptic clustering.

Loss of *PTEN*, a SFARI gene, can cause postsynaptic and presynaptic changes in excitatory and inhibitory connectivity. *In vitro* analyses have recently demonstrated that a miRNA (microRNA-301a) can downregulate *PTEN* after glycine receptor activation (Chen et al., 2016). Tao et al. (2019) showed that *miR-4465* significantly inhibited the expression of *PTEN*, upregulated phosphorylated AKT and ultimately inhibited autophagy by activating mTOR in HEK293, HeLa, and SH-SY5Y cells (Tao et al., 2019). Half dosage of *hsa-mir-4465* in our patient is expected to cause upregulation of *PTEN,* contributing to postsynaptic impairment. MiRNAs are important regulators of brain development and neuronal function, and have been associated with a variety of nervous system diseases, including ASD (Cheng et al., 2018; Wu et al., 2020).

In mice, the deletion of *Inpp5a* causes perinatal lethality in 90% of the homozygous mutants, early onset ataxia and relatively small stature in surviving mutants. Heterozygotes do not exhibit obvious motor coordination impairment unless challenged in motor skill test (Yang et al., 2015). *Inpp5a* is a downstream effector of signalling from mGlu1 receptor, one of the two members of group I metabotropic glutamate receptors, that regulates synaptic plasticity, particularly in cerebellar Purkinje cells. The mGlu1 receptor activity needs to be fine-tuned and balanced for normal motor coordination. Alteration of expression, either enhancement or decrease, of group 1 mGlu metabotropic receptors in mice is known to alter their activity, which results in defects of motor coordination (Rossi et al., 2013; Bossi et al., 2018). The deletion of *INPP5A* in this patient may be in part responsible for his impairment in motor skills.

**FIGURE 6:**
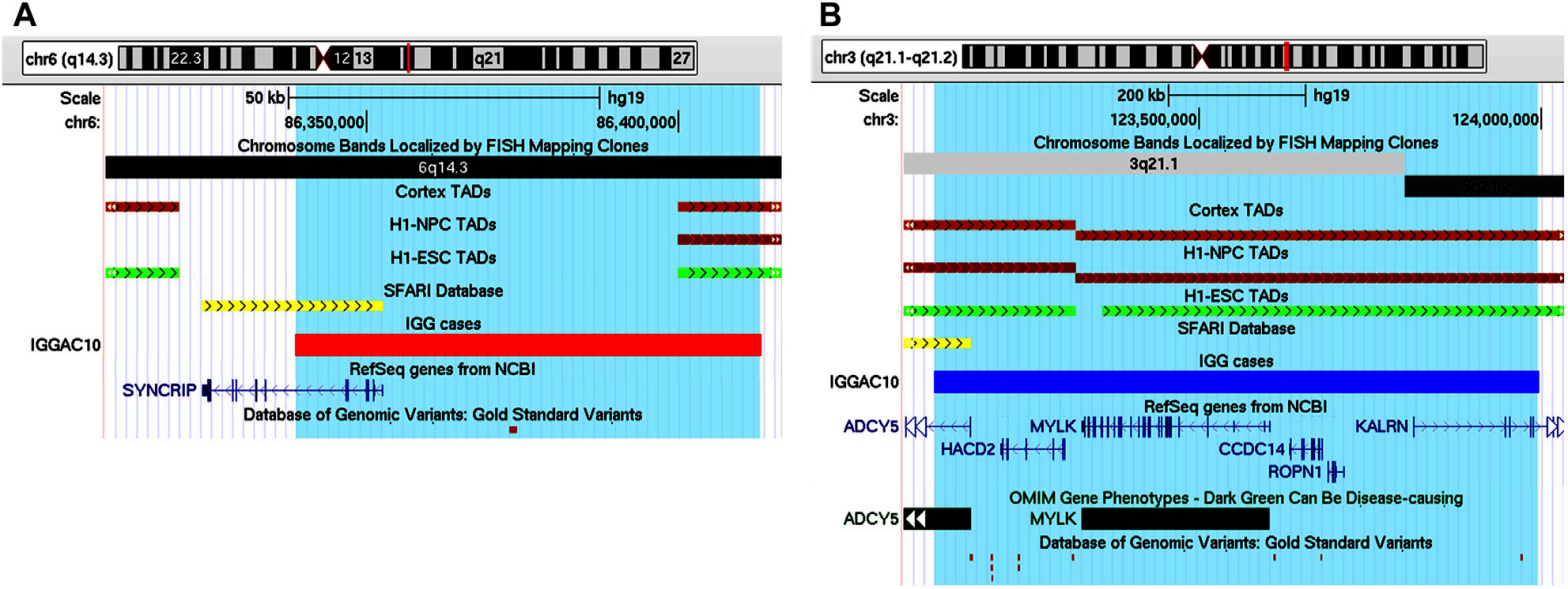
Additive effects between *SYNCRIP* and *ADCY5* in patient IGGAC10. **(A)** Screenshot of chromosome 6 region encompassing the deletion of patient IGGAC10 (red bar, chr6:86,338,564-86,413,285). TADs from Cortex, H1NPC and h-ESC cells. **(B)** Screenshot of chromosome 3 region encompassing the duplication of patient IGGAC10 (blue bar, chr3:123,113,523-123,997,093). TADs from Cortex, H1NPC and h-ESC cells.

IGGAC10 patient showed a complex phenotype mainly characterized by intellectual disability and epilepsy (Table 1). He carried a maternal deletion encompassing *SYNCRIP* and a paternal duplication involving the upstream regulatory genomic region of *ADCY5*. Both CNVs overlap a TDB between two flanking TADs described in different tissues, including brain cortex, which could also contribute to dysregulating the expression of implicated genes (Figures 6A,B).

*SYNCRIP* encodes the synaptotagmin-binding cytoplasmic RNA-interacting protein, a member of the cellular heterogeneous nuclear ribonucleoprotein (hnRNP) family that plays a role in multiple aspects of mRNA maturation. Missense and truncating variants of *SYNCRIP* were found in ASD patients with more severe phenotypes (Guo et al., 2019) and in patients with severe non-syndromic sporadic intellectual disability (Rauch et al., 2012), respectively. *ADCY5* encodes a member of the membrane-bound adenylyl cyclase enzymes which mediate G-protein-coupled receptor signalling through the synthesis of the second messenger cAMP. According to SFARI database, missense and frameshift variants of *ADCY5* have been reported in ASD patients. Gain of function variants of *ADCY5* have been associated with a broad range of movement disorders, most notably chorea, dystonia and myoclonus (Vijiaratnam et al., 2019).

Thus, the two CNVs could cause, by a mechanism also implicating a modification of chromatin conformation, a dysregulation of both *SYNCRIP* and *ADCY5* contributing to the complex phenotype observed in this patient.

**FIGURE 7:**
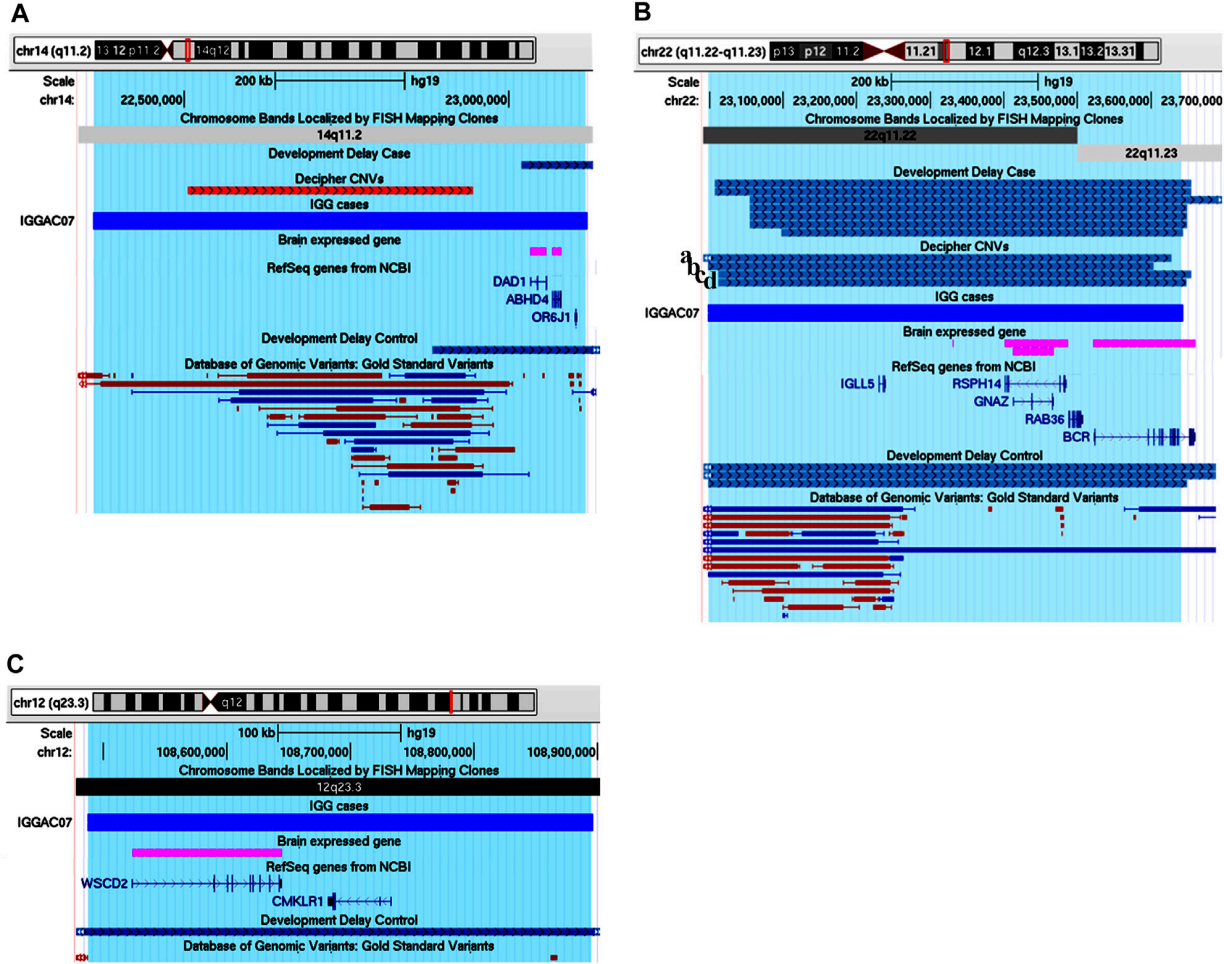
Additive effects of *ABHD4*, *GNAZ* and *WSCD2* in patient IGGAC07. **(A)** Screenshot of chromosome 14 region encompassing the duplication of patient IGGAC07 (blue bar, chr14:22,360,671-23,120,435). Brain-expressed genes (see *Methods*). **(B)** Screenshot of chromosome 22 overlapping the duplication of patient IGGAC07 (blue bar, chr22:22,998,284-23,643,223). Duplications in Decipher database (a: patient 251352; b: patient 286708; c: patient 301091; d: patient 288064). Brain-expressed genes (see *Methods*). **(C)** Screenshot of chromosome 12 overlapping the duplication of patient IGGAC07 (blue bar, chr12:108,487,109-108,896,252). Brain-expressed genes (see *Methods*).

IGGAC07 patient carried a *de novo* duplication of chromosome 14, and two maternally inherited duplications of chromosome 12 and chromosome 22, respectively (Table 2; Figures 7 A–C).

Interestingly, chromosome 14 duplication encompassed *ABHD4*, a gene recently shown to be involved in developmental anoikis, a mechanism of cell death in the prenatal brain preventing from survival of misplaced cells (Laszlo et al., 2020). Indeed, *ABHD4* is involved in N-acylethanolamine biosynthesis, including the endocannabinoid molecule anandamide which has various physiological functions such as the regulation of synaptic plasticity and apoptosis. *ABHD4* expression is tightly controlled spatially and temporally in the developing brain and a misregulation of its expression due to the duplication could contribute to the patient’s phenotype. Duplications encompassing *ABHD4* were annotated in only one control individual, while various cases were reported both in Decipher and in Developmental Delay databases with duplications larger than that observed in our patient and a smaller one sharing with our patient *ABHD4* duplication.

The maternal duplication on chromosome 22 involved a region with triplosensitivity including *GNAZ,* the Guanine Nucleotide-binding protein (G protein) Alpha Z polypeptide encoding gene. In this region, many duplications are reported in both Decipher and Developmental Delay databases, most of them extending over the short region containing *GNAZ*. Only a total of 4 control individuals are reported in either Developmental Delay Control or in DGV databases with duplications overlapping the *GNAZ* gene. *GNAZ* is a member of the Gαi subfamily of heterotrimeric G proteins, and couples to G-protein-coupled receptors triggering important pathways in neuronal development (Hultman et al., 2014). The duplication of chromosome 12 entirely encompassed *WSCD2*, which encodes a WSC Domain-containing protein with a still unclear function. Interestingly, genome wide association analyses found significant results for variants in *WSCD2* in patients with psychiatric disorders (Lo et al., 2017). Finally, we could hypothesise that all the *de novo* and the inherited duplications could have jointly contributed to the NDD phenotype of patient IGGCA07.

### CNV-Mediated Complex Pathogenic Mechanisms

We found 4 patients with CNVs that could affect expression of NDD genes throughout an indirect and/or long-range effect (Table 1 and Table 2).

**FIGURE 8:**
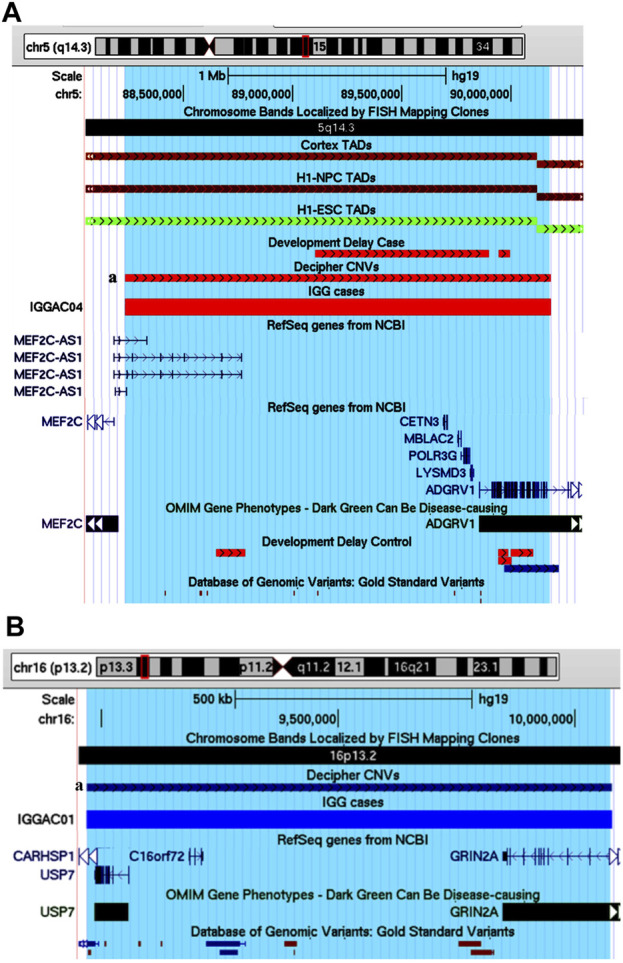
Potential candidate genes and pathogenetic mechanisms in patient IGGAC04 and IGGAC01. **(A)** Screenshot of chromosome 5 region overlapping the deletion of patient IGGAC4 (red bar, chr5:88,232,587-90,181,774). Decipher CNVs (a: deletion in patient 251716). TADs from Cortex, H1NPC and h-ESC cells. **(B)** Screenshot of chromosome 16 region overlapping the duplication of patient IGGAC01 (blue bar, chr16:8,969,984-10,078,802). Decipher CNVs (a: duplication in patient 382832).

One case (IGGAC04) presented with very severe psychomotor delay, hypotonia, and epilepsy (Table 1), carrying a *de novo* heterozygous deletion of chromosome 5 (Figure 8A). This deletion encompasses 6 genes, two of them expressed in the nervous system: *MEF2C-AS1,* which encodes a lncRNA annotated as antisense RNA of *MEF2C*, and *ADGRV1,* which encodes a member of the G-protein-coupled receptor superfamily associated with familial febrile seizures and Usher syndrome 2 (OMIM 602851). *MEF2C* (myocyte enhancer factor-2, OMIM 600662) is a transcription factor playing a role during development, mostly in myogenesis and neurogenesis. *MEF2C* haploinsufficiency has been shown to be responsible for severe cognitive deficit with stereotyped movements, epilepsy, and/or cerebral malformations (Le Meur et al., 2010). It has been recently demonstrated that, during myogenic differentiation a lncRNA, the *OIP5-AS1*, through its complementarity with *MEF2C* 3′UTR, enhances *MEF2C* mRNA stability thus promoting *MEF2C* expression and ultimately enhancing myogenesis (Yang et al., 2020). We hypothesized that during neurogenesis, analogously to *OIP5-AS1*, *MEF2C-AS1* could enhance *MEF2C* mRNA stability and promote *MEF2C* expression and neurogenesis. In this view, the heterozygous deletion observed in patient IGGAC04 could reduce the expression of *MEF2C-AS1* causing a decrease of *MEF2C*, thus interfering with neuronal differentiation and contributing to the patient’s phenotype. Although as a consequence of *MEF2C-AS1* deletion a gene loss-of-function is likely, functional experiments would be required to exclude some compensatory mechanisms, as up-regulation of the healthy allele. The deletion of *ADGRV1* could be responsible for the epilepsy phenotype. Of note, we found one patient in the Decipher database (Patient 251716) carrying a deletion overlapping the one of our patient and having similar clinical features characterized by intellectual disability, muscular hypotonia, and seizures. No deletions overlapping the same region were present among controls (Developmental Delay and DGV Controls). The chromosome 5 region deleted in these patients overlaps a TDB described in different tissues, including those relevant for neurodevelopment (e.g., Cortex), according to their relative Hi-C maps (Figure 8A), which could further complicate the fine regulation of expression of the overlapped genes, including *MEF2C-AS*.

One case (IGGAC01) showed a complex phenotype mostly characterized by psychomotor delay, autistic features, hypotonia, epilepsy, gastrointestinal and sleep disturbances (Table 1), and carried a *de novo* heterozygous duplication of chromosome 16 (Figure 8B). The duplication involved, in particular, two OMIM reported genes, *USP7,* encoding the Ubiquitin Specific Peptidase 7, and *GRIN2A,* encoding the NR2 subunit of the N-methyl-D-aspartate (NMDA) glutamate receptor. *USP7* has been shown to regulate the ubiquitination of a variety of proteins with functions in different biological processes as oxidative stress response, histone modification and regulation of chromatin remodelling (Khoronenkova et al., 2011). Deletions, truncating and missense variants of *USP7* have been associated with the Hao-Fountain syndrome (OMIM 616863), characterized by speech delay, autistic spectrum disorder, attention-deficit hyperactivity disorder, sleep disturbances and gastroesophageal reflux disease (Fountain et al., 2019). All these clinical features are present in our case who has the complete duplication of *USP7*. Notably, in syndromic forms of ASD, duplications in addition to deletions of *USP7* have been reported (Sanders et al., 2011), thus supporting the idea of dosage sensitivity, with both low and high *USP7* expression causing imbalances of neuronal homeostasis. The duplication present in patient IGGAC01 partially overlapped *GRIN2A*. Truncating as well as missense, activating variants of *GRIN2A* have been associated with an idiopathic form of focal epilepsy, EEG continuous spike-and-wave during sleep (CSWS), and speech disorder (OMIM 245570) (Lemke et al., 2013) whose clinical aspects are present in our patient. Different molecular alterations of NMDA receptor subunits seem to result in a deleterious dysregulation of NMDA receptor function (Balu and Coyle, 2011). Although the effect of partial duplications on gene function are not easily predictable, some clinical features of the IGGAC01 patient suggested the occurrence of a dysregulation of *GRIN2A* that could have contributed to the patient’s phenotype. Of note, a patient with phenotype resembling that of patient IGGAC01 was reported in Decipher database with a similar duplication, including both *USP7* and *GRIN2A* (Figure 8B). We thus hypothesised that the phenotype of these two patients could result from the impairment of both *USP7* and *GRIN2A.*

**FIGURE 9:**
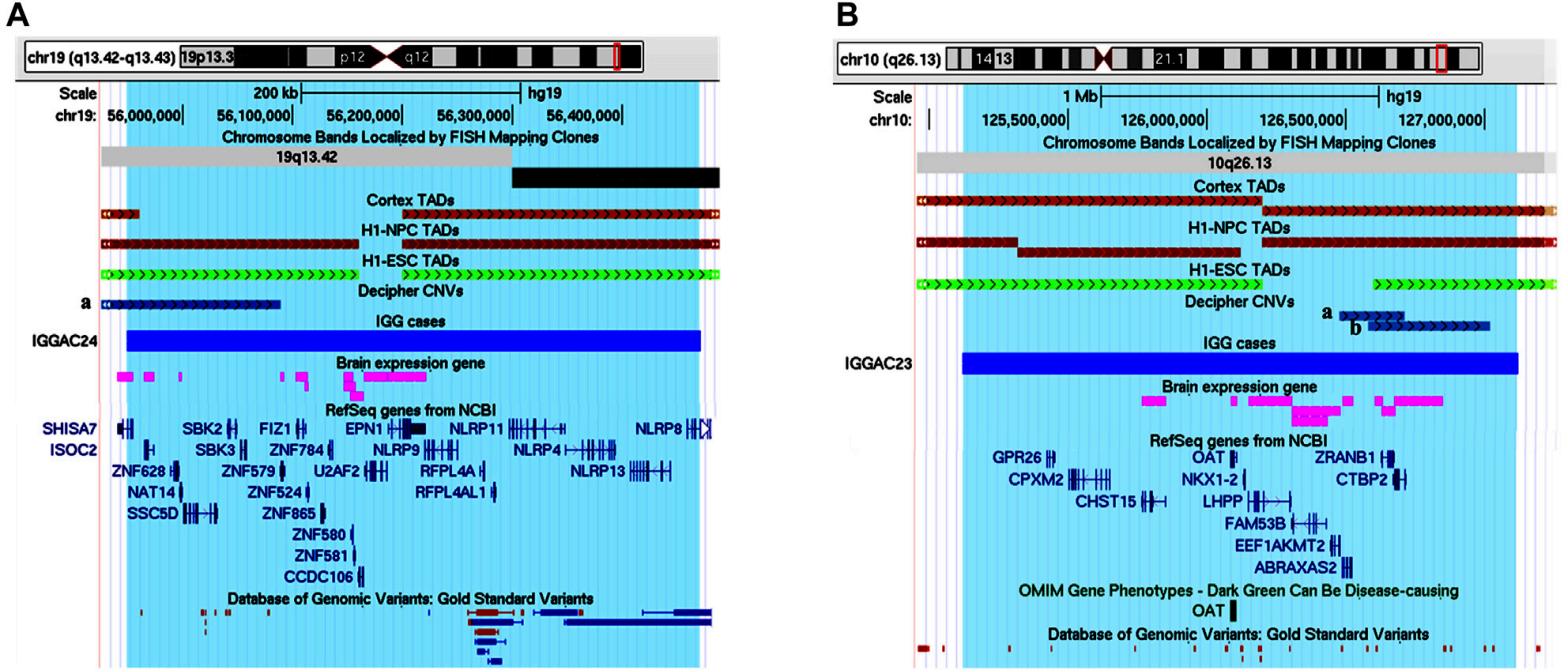
Duplications of topological domain boundaries (TDBs) potentially involved in the phenotype of patient IGGAC24 and patient IGGAC23. **(A)** Screenshot of chromosome 19 region encompassing the duplication of patient IGGAC24 (blue bar, chr19:55,948,706-56,471,996). TADs from Cortex, H1NPC and h-ESC cells. Brain-expressed genes (see *Methods*). Decipher patient with reported duplication (a. patient 338729) **(B)** Screenshot of chromosome 10 region encompassing the duplication of patient IGGAC23 (blue bar, chr10:125,121,038-127,119,447). Brain-expressed genes (see *Methods*). Decipher patients with reported duplications (a: patient 283429, b: patient 378968).

IGGAC24 had a *de novo* duplication of chromosome 19 that encompasses various genes, 11 of which are expressed in the brain. The duplication also overlaps a TDB described in the brain cortex and other different tissues, which could contribute to dysregulating gene expression of implicated genes (Figure 9A and Table 1)*.* Attempting to prioritize enclosed genes and define possible candidate genes, we performed a gene enrichment analysis as described above (see *Methods*). Two genes emerged for their functions known to play a role in neurodevelopmental disorders, namely *SHISA7* and *U2AF2*. Indeed, in Decipher at least one NDD case with a duplication that partially overlaps the TDB and the *SHISA7* gene (Patient 338729**)** was reported while no similar duplications, implicating brain-expressed genes, were reported among controls. We focused on *SHISA7*, a transmembrane protein that interacts with GABAA receptors (Han et al., 2019) and, by acting as auxiliary protein for the trafficking of AMPA receptors, regulates synaptic plasticity (Schmitz et al., 2017). Notably, spine loss has been observed in *SHISA7* knockout mice and in wild type mice with *SHISA7* overexpression (Schmitz et al., 2017; von Engelhardt, 2019). Thus, we hypothesised that the duplication could cause a dysregulation of *SHISA7* expression, possibly also by altering chromatin conformation, and ultimately causing a dysregulation of synaptic plasticity.

Patient IGGAC23 showed a *de novo* duplication of chromosome 10 overlapping 7 brain-expressed genes and a TDB described in the brain cortex and other tissues (Figure 9B and Table 1). After gene enrichment analysis of these 7 genes with known NDD genes, one emerged as the most interesting, *CTBP2*. This gene produces two alternative transcripts for two different proteins, one of them is a component of specialized synapses (ribbon synapses) and the other one is a negative regulator of transcription. *CTBP2* knock-out mice present a neurological phenotype characterized by abnormalities of brain development. A recent study reported that the overexpression of *CTBP2* inhibits neuronal differentiation (Zhang et al., 2020). Of note, while no duplications similar to that of this patient were reported among controls, in Decipher a patient with autistic behaviour and a duplication shorter than the one of patient IGGC23, but still enclosing *CTBP2*, was reported. Thus, the duplication present in patient IGGAC23 could have caused overexpression and/or dysregulated expression of overlapped genes, among which *CTBP2*, whose overexpression has already been associated with impaired neuronal defects.

## Discussion and Conclusion

In the frame of identifying the most likely genes and genetic mechanisms underlying complex phenotypes in neurodevelopmental disorders, and to better interpret CNV causal effects in complex cases, we performed a detailed analysis of both clinical features and CNVs of 12 patients.

In particular, we analyzed patients’ CNVs in the light of information on gene expression, gene function, and chromatin organization features of involved genomic regions. Then we assessed the presence of potential deleterious CNVs acting in concert in the same patients, according to a two-hit model emerging from the recent literature as a possible pathogenetic mechanism with important roles in neurodevelopmental disorders.

Four patients reported with a complex phenotype, i.e. not referable to a known syndromic neurodevelopmental disorder, carried a CNV that can possibly have indirect deleterious effects on neuronal expressed genes and finally on their phenotype. In one case, a deletion encompassed *MEF2C-AS1*, the antisense gene of a known NDD-associated gene, *MEF2C*. Involvement of antisense genes in NDDs has been rarely reported so far. One example is represented by *PTCHD1-AS*, the antisense-gene of the NDD-associated gene *PTCHD1*. In fact, although the molecular function of *PTCHD1-AS* is still to be clearly elucidated, it has been demonstrated that its disruption diminished miniature excitatory postsynaptic current frequency in iPSC-derived neurons from subjects with ASD, thus supporting a role for this long noncoding RNA in the etiology of ASD (Ross et al., 2020). Interest on regulatory elements of gene expressions, like lncRNA and regulatory enhancer elements, and their involvement in brain disorders has been recently increased and new tools have been applicated for their detection, as those implicated in ASD and Schizofrenia (Piluso et al., 2019; Alinejad-Rokny et al., 2020).

Deletions and duplications may overlap TDBs between two flanking topological associated domains causing a dysregulation of chromatin organization and, in turn, a dysregulation of implicated genes which ultimately affects the patient’s phenotype. So far, implication of CNVs in TAD boundaries have been rarely investigated in NDDs (Di Gregorio et al., 2017; Melo et al., 2020). Chromatin conformation domains can be different in various cells and tissues, and we were aware that effects of deletions and duplications deduced on the basis of TDBs data reported in databases have to be considered hypothetical, deserving further experimental validation. To tentatively achieve a more reliable data analysis of our patients, we considered only TDBs reported in more than one tissue, assuming that they could possibly represent a chromatin feature common to many tissues, including brain areas involved by NDDs. All our four cases have a CNV encompassing a TDB between two flanking topologically associated domains described in at least three tissues which could have played a role in NDD phenotype, such as brain cortex (Schmitt et al., 2016) and human embryonic stem cell-derived neurons (Dixon et al., 2015).

One important finding emerging from this evaluation of diagnostic array-CGH results is the presence of co-occurring CNVs that, according to the two-hit model, could have contributed to the patients’ phenotype.

In eight patients, we found two CNVs overlapping either known NDD genes and/or genes with potential roles in neurodevelopment. In three patients, we found one *de novo* and one variant inherited from unaffected parents, the remaining five patients inherited one CNV from each of their unaffected parents. In three out of eight cases, namely IGGAC06, IGGAC07, and IGGAC08, involved genes have not yet been associated with disease and we presented data in favor of their implication in neurodevelopmental disorders. Indeed, we searched the Decipher database for cases with one CNV corresponding to that presented by our patients and a secondary CNV, involving genes with a role in neurodevelopment. We found some cases, reported in the figures, that further support the hypothesis that identified co-occurring CNVs in our patients act additively causing deleterious phenotypes according to a two-hit model.

**FIGURE 10:**
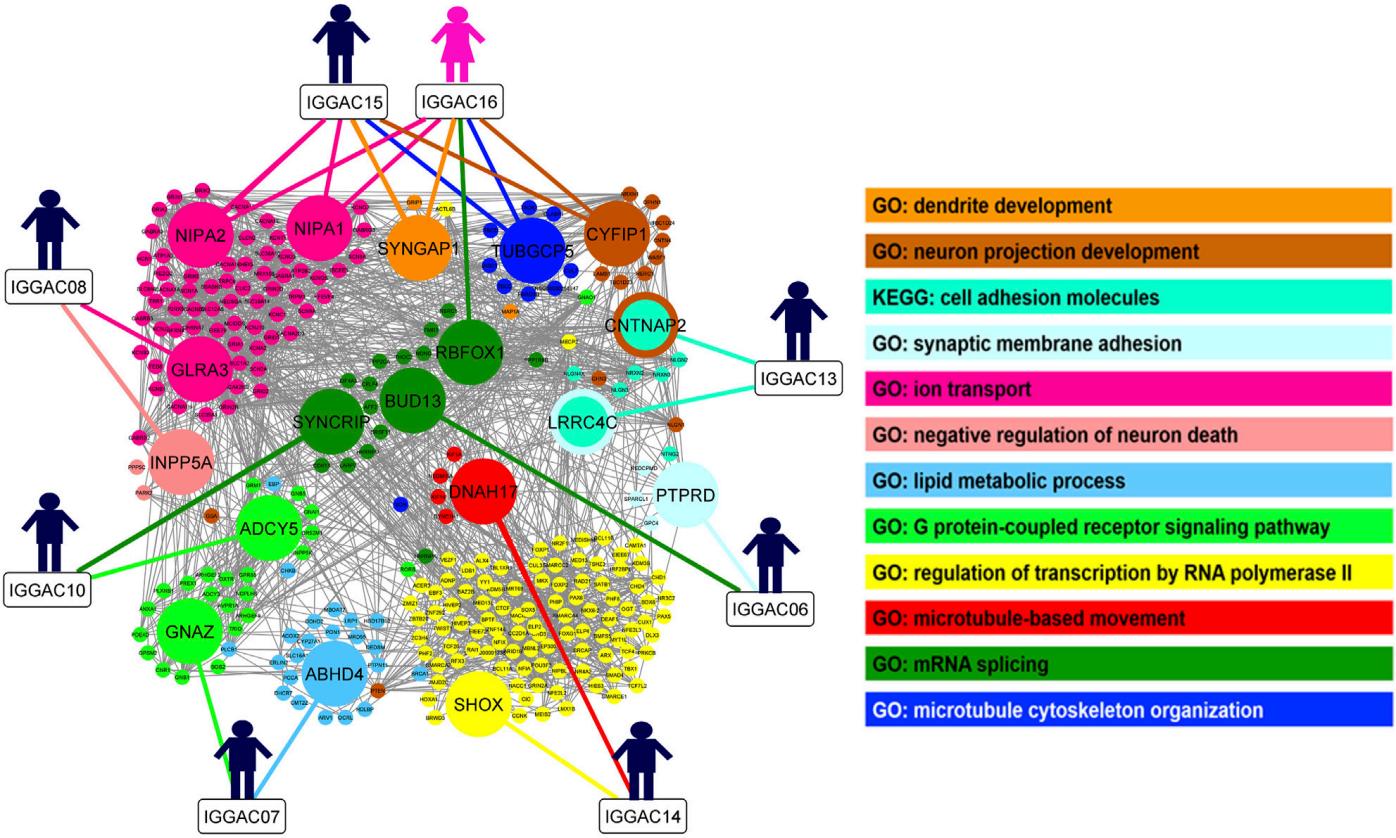
Overview of biological processes and pathways involved in patients with CNV-genes acting according to double-hit model. The network depicts the protein-protein interactions (grey lines) revealed by STRING analysis (visualized by Cytoscape tool) on the basis of the enrichment obtained with GeneCodis4 tool. The Figure shows the enriched BP terms and KEGG pathways clustering the CNV-genes of the eight patients with known NDD genes (SFARI reported genes with score 1/2 and OMIM genes correlated to NDDs). The analysis shows that all eight patients have CNV-overlapped genes involved in two known NDD-associated biological processes, except patient IGGAC13, for whom the two implicated genes appeared in the same KEGG pathway. GO terms “neuron projection development”, “synaptic membrane adhesion”, “ion trasport”, “G-protein-coupled receptor signalling pathway” and “mRNA splicing” are shared by at least two different patients.

In order to gain insight into possible interaction among genes implicated in the CNVs co-occurring in the same patient and to evaluate whether these genes share molecular functions or biological processes, we performed gene enrichment and protein-protein interaction analyses (Figure 10; Supplementary Table S1). We also tested interaction with other known NDD associated genes, which helped to better define the role of new candidate genes in important neurodevelopmental pathways and biological processes. In one case, IGGAC13, the two major implicated genes, i.e., *CNTNAP2* and *LRRC4C*, interact with each other through *NRXN1* and with other NDD-associated genes and have a common function in the KEGG cell adhesion molecule pathway (Figure 1). In all the other cases, genes affected by co-occurring CNVs do not seem to directly interact, but they have a role in important, although distinct, biological processes that together contribute to proper brain development (Figure 10). We thus hypothesized that, in all these cases, the double-hit mechanism by affecting two pathways/biological processes finally determines an impairment of neurological development.

As observable in Figure 10, couple of patients have genes involved in the same pathway/biological process and one additional gene dealing with another biological process. As an example, both IGGAC06 and IGGAC10 have one CNV each involving a different gene but with a common function in mRNA splicing, and each of the two patients has an additional CNV involving one gene with a role, respectively, in synaptic membrane adhesion and in G-coupled receptor signaling, two fundamental functions of synapse.

Of note, co-occurring secondary CNVs overlapping NDD genes were also found in patients with syndromic or recurrent gene-specific CNVs, which could modulate penetrance and/or severity of the disease. This phenomenon is well exemplified by the case of two siblings, IGGAC15 and IGGAC16, who shared two CNVs overlapping known NDD genes, including an already described deletion of chromosome 15 with reduced penetrance (Cox and Butler, 2015). In addition to these two CNVs, patient IGGAC16 also carried a deletion of chromosome 16 implicating *RBFOX1*, a known NDD gene, also described with reduced penetrance (Lal et al., 2013). Patient IGGAC16, the one of the two siblings who carried a total of three CNVs, exhibited a more complex phenotype compared to her brother.

Overall, our genetic analysis allowed to unveil candidate genes and to infer some synergistic and complex mechanisms that could origin the neurodevelopmental disorder in these patients. However, novel candidate genes and genes affected by co-occurring CNVs although individually associated with NDD biological processes, require further functional analyses to understand their potential interactions and ultimately to clarify their contribution to patient phenotypes.

Variability and complexity of genetic components make the functional validation of NDD genes and pathogenic mechanisms a challenge. Validation of hypothesised mechanisms in one patient can represent a research project by itself. Concerning the present cases, we could consider different levels of validation starting from verifying the effect of a CNV on expression of the implicated genes in induced neurons (iN) that could be obtained from patients’ fibroblasts. With this approach we could evaluate the effects of deletions of antisense or miRNA genes on the expression of their specific targets (as for patient IGGAC4 and IGGAC8) or of CNV involving TDB boundaries on implicated genes (as for patient IGGAC10). A further level of investigation is represented by the analysis of morphology and connections of iNs, usefull to investigate impairment of cell adhesion molecules, as for the case IGGAC13. Patient-derived iN could be used to evaluate defects on RNA splicing as in the case of IGGAC6 patient. To evaluate the effects of more complex genetic mechanisms on brain development and on behaviour, a further level of validation can be represented by studies with animal models, as mice. Indeed, as mice with double/multiple heterozygous variants can be obtained (Bossi et al., 2018), the synergistic effects of multiple variants could be also evaluated. In this view, and as mouse models for both *LRRC4C* and *CNTNAP2* genes are available, it could be interesting to obtain a mouse strain carrying heterozygous, loss of function mutations in both genes, thus mimicking the genotype of patient IGGAC13.

Our findings further support the hypothesis that double-hit mechanisms, involving inherited and *de novo* CNVs, could synergistically cause, or modulate, the neurodevelopmental phenotype. Indeed, the co-occurrence of pathogenic variants has also been observed after analysing the CNVs together with the results of whole exome sequencing, underlining the importance of the genetic background for the penetrance of a deleterious variant (Pizzo et al., 2019).

Recently reported data indicate that, in NDDs, WES analyses have diagnostic yields of about 36% while chromosomal microarray analysis of less than 20% (Srivastava et al., 2019; Savatt and Myers, 2021). As variants causing NDDs can be CNVs or single nucleotide variants (SNVs), isolated or even combined, the genetic diagnostic approach has to take into consideration methods able to detect both type of variants. Indeed, several clinical laboratories are now incorporating CNV calling into WES and are able to detect multi-exon deletions and duplications (Savatt and Myers, 2021).

WGS, by definition, detects more comprehensively all classes of genetic variants and, as cost decreases, will eventually supersede WES and chromosomal array analyses in clinical testing algorithms over time.

Finally, our work describes a possible integrated framework of investigation of CNVs based on publically available resources of genomic annotations and bioinformatic analyses to prioritize candidate genes and infer pathogenic mechanisms. This approach could be used for interpretation of those CNVs that, after a first level analysis, remains of uncertain significance.

Indeed, it would be interesting to test these patients by WES to check for single nucleotide variants which, according to multi-hit model, could further contribute to their phenotype.

## Data Availability

The datasets presented in this study can be found in online repositories. Clinical and genetic variant details have been deposited in the Decipher database with the following accession numbers: 433116 (IGGAC01); 433118 (IGGAC04); 433122 (IGGAC06); 433123 (IGGAC07); 433125 (IGGAC08); 433130 (IGGAC10); 433133 (IGGAC13); 433135 (IGGAC14); 433138 (IGGAC15); 433139 (IGGAC16); 433172 (IGGAC23); 433173 (IGGAC24).
