# Supplementary material for: Neurodevelopmental Disorders in Patients With Complex Phenotypes and Potential Complex Genetic Basis Involving Non-Coding Genes, and Double CNVs
Source: Front Genet. 2021 Sep 21;12:732002. doi: 10.3389/fgene.2021.732002 (PMC8490884; doi:10.3389/fgene.2021.732002)
Supplement: Supplementary file 1 [file DataSheet1.PDF]

Table S1. Biological processes and KEGG pathways associated with genes involved in the present cases with multiple CNVs and genes reported in the SFARI database and/or in OMIM as associated to neurodevelopmental disorders.

Annotation	Term	Genes found	Input size	Term genes	Genes universe	pval	pval_adj	Genes
GO:0016358	dendrite development	6	887	37	61686	0,00001381	0,00018191	GRIP1,**SYNGAP1**,MECP2,MAP1A,PPP1R9B,ACTL6B
GO:0007186	G protein-coupled receptor signaling pathway	27	887	682	61686	0,00000311	0,00005263	PLCB1,ARHGEF9,**ADCY5**,ADCY3,LADCI,NDPLHS,NEDMHM,TRIO,NS9,RORB,AVPR1A,PLXNB1,ACRDYS2,OXTR,ANXA1,SCA44,MRD42,**GNAZ**,NEDIM,GNAI1,OR52M1,**GPR123**,PREX1,COCPMR,MDCCAID,CMCS,CNR1
GO:0006811	ion transport	68	887	594	61686	0,00000000	0,00000000	MRXS32,MRXSRC,LKPAT,HMNDYT2,CHRNA7,SLC9A6,SCA42ND,CACNA1H,CACNB2,CACNA1E,CACNA1D,CACNA1C,CACNA1A,**GLRA3**,TRPC6,SLC1A2,SCN9A,SCN8A,FFEVF4,SCN2A,SCN1A,IECEE3,ATP2B2,AHC2,P2RX5,TRPM1,KCNS3,KCNQ3,KCNQ2,KCNJ10,KCNJ2,EPM7,KCNB1,MCIDDS,EIEE32,EIEE46,GRIN2B,GRIN2A,GRIN1,GRIK5,GRIK2,SCAR18,GRID1,NEDSGA,GRIA2,GRIA1,HCN1,GABRG3,FEB8,GABRB3,GABRB2,EIEE79,GABRA3,EIEE78,EIEE19,SLC38A10,**NIPA1**,COXPD18,MRX108,SSASKS,**NIPA2**,CDG2N,DA3,ENFL5,SLC12A5,MRD46,CACNA2D3,FHEIG
GO:0006629	lipid metabolic process	26	887	585	61686	0,00000057	0,00001162	DDHD2,CHKB,PLCB1,CMT2Z,SPG18,MEND,CBAS6,FANCS,MCT1D,PTPN11,PTEN,PON1,PCCA,OCRL,LRP1,HDLBP,HSD10MD,GBA,MRD55,DEDSM,MBOAT7,EIEE38,**ABHD4**,DHCR7,CYP27A1,PLDECO
GO:0000226	microtubule cytoskeleton organization	11	887	136	61686	0,00000488	0,00007692	CLASP1,CFEOM3A,CUL7,PEAMO,PEBAT,SON,MAP1A,PARD3B,**TUBGCP5**,TAOK1,DISC1
GO:0007018	microtubule-based movement	6	887	74	61686	0,00070194	0,00470892	SMALED2B,KIF14,**DNAH17**,MRD9,NEDMIBA,DYNC1H1
GO:0008380	mRNA splicing	18	887	264	61686	0,00000007	0,00000188	**SYNCRIP**,DDX48,SRSF11,CDK13,SON,PPP2CA,MRXS34,HNRNPU,AUKS,AFF2,FMR1,**BUD13**,PPP1R9B,MRX12,CELF4,**RBFOX1**,ALAZS,RSRC1
GO:1901215	negative regulation of neuron death	5	887	63	61686	0,00214674	0,01103270	PPP5C,PRKN,**INPP5A**,GBA,GABRB2
GO:0031175	neuron projection development	17	887	129	61686	0,00000000	0,00000000	**CNTNAP2**,**CYFIP1**,NLGN1,NRXN1,WASF1,MDFPMR,CHN3,PTEN,OPHN1,MECP2,LAMB1,NEDIM,GBA,CNTN4,PPP1R9B,FIME,TBC1D23

GO:0006357	regulation of transcription by RNA polymerase II	103	887	1111	61686	0,00000000	0,00000000	ZBTB20,NIPBL,ADNP,MED13L,EIEE67,CANPMR,ZC3H4,CIC,PHF8,SIHIWES,CHD3,CHD2,PILBOS,KDM4C,MYT1L,ZNF292,SRCAP,KDM5B,RAI1,TBR1,CTCF,DEAF1,MED13,NFE2L3,LDB1,CCNK,MRX106,CUL3,VEZF1,GDACCF,NEDISHM,CUGS,YY1,SWCOS,BMFS5,BBSOAS,TCF20,TCF7L2,TCF4,FANCS,TBX1,SOX5,CSS5,SMARCC2,SMARCA4,SMARCA2,**SHOX**,SATB1,RORB,RORA,RFX3,RAD21,PRKCB,POU3F3,PHF2,PAX6,PAX5,NR4A2,NFIX,MACID,IMDDHH,BRMUTD,EIEE72,NR3C2,MEIS2,MECP2,SMAD4,LMX1B,HOXA1,AUKS,HIVEP2,MKX,BRWD3,EBF3,ARX,FOXG1,NEDDFL,NACC1,FOXP2,EP300,HIES3,**NKX62**,TBL1XR1,IMD49,IRF2BPL,DLX3,FND2,HIVEP3,TSHZ3,ARID1B,ZMIZ1,SOX6,MRT58,PHIP,CUX1,CC2D1A,BCL11A,KDM3B,ACTL6B,BAZ2B,MRT69,FOXP1,ELP4
GO:0099560	synaptic membrane adhesion	6	887	23	61686	0,00000071	0,00001414	NLGN1,SPARCL1,**PTPRD**,GPC4,NTNG2,**LRRC4C**
hsa04514	KEGG: Cell adhesion molecules	12	887	63	61686	0,00000000	0,00000000	**CNTNAP2**,NEDCPMD,NLGN1,NRXN2,NRXN1,NRXN3,CHN3,NTNG2, **LRRC4C**,NLGN2,NLGN4X,NLGN3

Genes found: number of annotated genes in the reference list; Input size: total number of genes in the input list, including the genes involved in the present cases, SFARI reported genes and genes reported in OMIM as associated to NDDs; Term genes: number of annotated genes in the reference list; Genes universe: total number of human reference genes; pval: hypergeometric test pValue; pval_adj: corrected hypergeometric pValue using FDR procedure.
